# The Safety of Contraction of Subcutaneous Tissue Following Liposuction Procedures

**DOI:** 10.1093/asjof/ojad112

**Published:** 2023-12-20

**Authors:** Sachin M Shridharani, Paul G Ruff, Vaishali B Doolabh, Edward M Zimmerman

## Abstract

This paper examines the practice of using a helium plasma radiofrequency (RF) device for contracting subcutaneous soft tissue following liposuction in all body areas. A review of the data from 6 industry-sponsor-initiated retrospective studies was performed, wherein 483 real-world patients underwent liposuction followed by contraction of the subcutaneous soft tissue with the helium plasma RF system. These data were evaluated to determine if any new or increased risks were introduced compared to the risks of liposuction alone. The totality of the real-world data demonstrates there are no new or increased risks for helium plasma RF procedures following liposuction compared to liposuction alone. These data support the safety of helium plasma RF for subcutaneous soft-tissue contraction following liposuction. There are currently no alternative therapies specifically cleared by the FDA that can claim use following liposuction for the purpose of contracting the subcutaneous soft tissue.

**Level of Evidence: 3:**

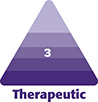

Liposuction can dramatically improve facial and body contours through the removal of excess, unwanted fat but it does not contract the subcutaneous tissue (Figure 1). Removal of significant fat volume in 1 liposuction procedure often surpasses the body's natural ability to contract the overlying skin/soft tissue, leaving many patients with undesirable dermal laxity. This sagging or drooping skin is a result of skin- and soft-tissue-dependent positioning, gravitational descendant, and/or postural redraping. Even with the introduction of energy during liposuction, such as laser-assisted liposuction (LAL) or ultrasound-assisted liposuction, many patients are left with undesirable skin laxity.^1-4^ To address the loose skin, physicians may choose to perform invasive, excisional procedures that carry more risks and comorbidities, such as increased blood loss, longer healing times, increased risk of infection, and larger scar areas.^5^ For the patient, excisional surgery increases the postoperative and self-care requirements, and patient recuperation time is increased significantly.

**Figure 1.**
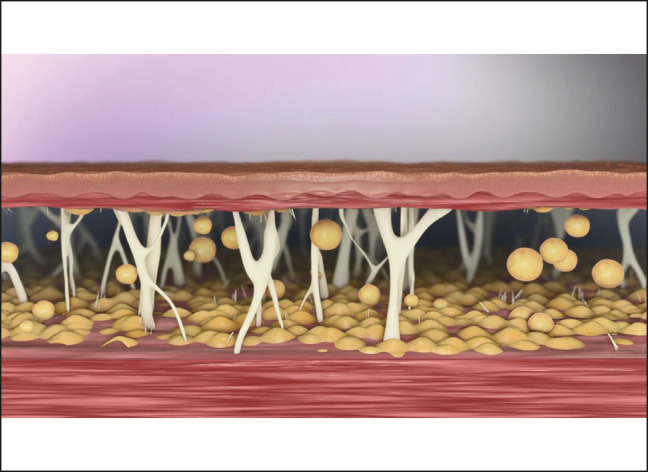
For illustrative purposes, subcutaneous connective tissue and fat after liposuction. Image provided by Apyx Medical with permission for use in this publication.

There are noninvasive procedures that can be performed to reduce skin laxity. These noninvasive devices involve the trans-epidermal delivery of ultrasound, light, or radiofrequency (RF) energy. However, these devices only provide modest outcomes compared to excisional procedures.^1,4,6^

The principles of thermally induced contraction of collagen through denaturation, and the contraction of soft tissue are well-established in the scientific literature in relation to the functional performance of minimally invasive (eg, LAL and RF) electrosurgical devices.^7^ The devices that work on the principle of bulk tissue heating have proven effective in reducing skin laxity; however, the process of heating and maintaining the required tissue temperature needed for maximal collagen contraction to occur is time-consuming and requires monitoring of epidermal temperatures since the length of time heat delivery is required can negatively affect the epidermis.^7-14^

Conversely, the Renuvion helium plasma RF handpiece (Apyx Medical, Clearwater, FL) heats the soft tissue to approximately 80°C to 90°C and causes contraction and denaturation of the collagen structure after only 0.04 s (Figure 2).^15^ Tissue heated between 60°C and 100°C undergoes protein denaturation, where hydrothermal bonds between protein molecules are instantaneously broken and then quickly reform as the tissue cools. This leads to the formation of a uniform coagulum (clumps of protein). During this process, the protein denaturation (breaking of the hydrothermal bonds) causes the tissue to contract.^15^ Heating the subcutaneous tissue results in the tissue effect of contraction which causes the subcutaneous tissue to contract which pulls the skin closer to the muscle which results in contraction of the skin. Furthermore, as the tip of the helium plasma device is drawn through the subdermal plane, new tissues are introduced to the energy which allows the plasma beam to quickly alternate between treating the different tissues surrounding the tip of the device. The tissue surrounding the treatment locations remains at cooler temperatures compared to bulk tissue heating devices, resulting in rapid cooling after the application of the energy through conductive heat transfer. In the process, less heat is transferred to the epidermis, resulting in safe external temperatures without the need for epidermal temperature monitoring (Video, available online at www.asjopenforum.com).

**Figure 2.**
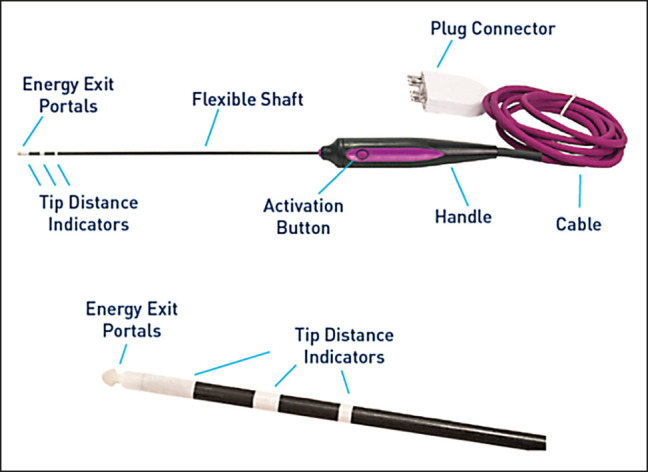
Renuvion APR Handpiece. Image provided by Apyx Medical with permission for use in this publication.

The benefit of this reduction in tissue volume was demonstrated in a study completed by Ibrahiem et al where an 8.8% reduction in the need for revision procedures involving the excision of excess tissue was observed for patients undergoing liposuction followed by helium plasma RF when compared with patients undergoing liposuction alone.^16^ Additional analyses were needed to evaluate if any new or increased risks were introduced when adding a helium plasma RF procedure following liposuction compared to the risks of liposuction alone. Whereas other minimally invasive products have *general* clearances for electrocoagulation and hemostasis, the helium plasma RF device has clearance specifically for contraction of subcutaneous soft tissues following liposuction for aesthetic body contouring. Data obtained from this review were used in support of this FDA indication.

This paper aims to summarize data collected from 6 previous industry-sponsor-initiated retrospective studies and evaluate any additional risks from the use of the helium plasma RF handpiece post-liposuction compared to liposuction alone. This review sought to answer the following questions: Are adverse events (AEs) any higher when you use helium plasma RF after liposuction compared to liposuction alone? Are AEs higher in any particular body areas? Do the device settings need to be different for different body areas?

## METHODS

Six separate previously published^17-21^ retrospective studies that collected data from the medical records of clinical sites where helium plasma RF was used following liposuction were evaluated (Table 1). These retrospective studies were conducted between September 2018 and May 2021. The data analysis of the combined retrospective study data was conducted from December 2022 to January 2023. Retrospective studies where patients did not have liposuction performed prior to helium plasma RF were excluded from this analysis (eg, dermal resurfacing or helium plasma RF-only procedures). Further, any patients who were identified as having data in any other of the original retrospective studies were also reported as screen failures in the second study for the purpose of this analysis, so as not to duplicate patient data.

**Table 1.** Included Patients From 6 Published Helium Plasma Studies

Study Protocol no.	Enrolled patients	Screen failures/Exclusions^a^	Total patients included
VP-1783	152^17^	4	148
VP-1910	192^18^	43	149
VP-1801	37^17^	28	9
APX-20-02	84^19^	1	83
APX-21-03	49^20^	1	48
APX-21-01	47^21^	1	46
TOTALS	561	78	483

^a^Data related to expected clinical side effects as defined in the IFU for the product were outside the scope of this analysis as these are expected treatment effects for both helium plasma RF and liposuction procedures. Adverse events outside the liposuction and helium plasma RF treatment areas were also not included in the analysis.

Demographics, procedure data, and AE data were reported. Data related to expected clinical side effects as defined in the instructions for use (IFU) for the product (discomfort/pain, edema, erythema, ecchymosis, hypoesthesia, touch sensitivity, itching, temporary weight gain, temporary numbness/tingling, transient migratory firmness, temporary, and/or transient crepitus) were outside the scope of this analysis, as these are expected treatment effects for both helium plasma RF and liposuction procedures. AEs outside the liposuction and helium plasma RF treatment areas were also not included in the analysis. Clinical side effects that were not expected and that were identified in the original retrospective studies by the investigators as AEs were included in the analysis.

Collected AEs were compared to published rates provided by Halk et al^22^ in their systematic review of safety studies in the field of liposuction. Additionally, treatment settings and AEs were analyzed by body area to determine if a particular body area increases the risk for AEs in liposuction procedures followed by helium plasma RF over and above the risk of liposuction alone.

The analyses described in this paper refer to data as “real-world data (RWD)” or “real-world evidence (RWE).” These terms and this data analysis follow the FDA guidance “Considerations for the Use of Real-World Data and Real-World Evidence to Support Regulatory Decision-Making for Drug and Biological Products,”^23^ whereas “RWD are data relating to patient health status and/or the delivery of health care routinely collected from a variety of sources” and “RWE is the clinical evidence about the usage and potential benefits or risks of a medical product derived from analysis of RWD.”^23^

Additional ethical approval was not required, as this analysis was based on data previously collected in IRB-approved retrospective chart reviews. No new data was gathered for this data analysis.

## RESULTS

Of the 483 patients included in this analysis who were treated with helium plasma RF following liposuction, 71% were females and 29% were males. Mean age was 44.7 ± 12.2 (range, 20-84). BMI was 27.2 ± 5.0 (range, 17.7-47.4).

### Are Adverse Events (AEs) Higher When Using Helium Plasma Radiofrequency After Liposuction Than Liposuction Alone?

The RWD in this analysis provides evidence that there are no increased risks to patients using helium plasma RF after liposuction compared to published data from patients having only the liposuction performed. There were no deaths, serious AEs, embolisms, significant bleeding events, or infections documented in this RWD analysis. A total of 32 AEs (0.07%) were captured in the 483 patients analyzed (Table 2). Patients were treated in the following body areas: abdomen, arms, back, buttocks, breast/axilla, face, hips/flanks, legs, and neck. One patient experienced epidermolysis which is classified as “burn/skin necrosis/blister,” 4 patients experienced hematomas and 18 patients experienced seromas; these are combined in this analysis for a rate of 4.6% (22/483) for “hematoma/seroma” to allow for a direct comparison to the data published by Halk for liposuction alone. Two cases of temporary nerve changes were experienced in this data review—1 patient experienced temporary motor nerve weakness of the marginal mandibular nerve (MMN) after liposuction followed by helium plasma RF in the neck that fully resolved without intervention and another patient experienced temporary hypoesthesia/numbness after liposuction followed by helium plasma RF in the face that also fully resolved without intervention.

**Table 2.** Adverse Events for Liposuction Alone and Liposuction Followed by Helium Plasma RF

Adverse event category	Published rates for liposuction alone (*n* = 537-496,245)	Patient rates for liposuction followed by helium plasma RF (*n* = 483)
Death	0%-0.06%	0%
Serious adverse events	0%-2.19%	0%
Embolism	0%-0.05%	0%
Significant bleeding	0.01%-0.23%	0%
Burn/skin necrosis/blister	0%-2.38%	0.2% (1)
Hematoma/seroma	0.03%-35.02%	4.6% (22)
Infection	0.01%-0.34%	0%
Temporary nerve changes	Not reported	0.4% (2)
Wound-related problem (scarring, inflammation, fibrosis, induration, nodule, open wound)	0.02%-1.99%	1.4% (7)

One patient experienced delayed healing, 2 patients experienced subcutaneous induration, 2 patients experienced subcutaneous nodules, and 2 patients experienced wound complications; for the sake of this analysis, these are all combined into “Wound-Related Problem” for a rate of 1.4% (7/483).

### Are AEs Higher for any Particular Body Area?

The AE data were also analyzed by body area. The RWD in this analysis provide evidence that there are no increased risks for any particular body area when performing helium plasma RF following liposuction over and above liposuction alone (Table 3). This analysis utilized a conservative approach where AEs are associated with each body area treated when it was not possible to associate the AE with a particular body area. For example, if a patient who received treatment in 3 different body areas experienced subcutaneous induration and the location of the subcutaneous induration was not specified, the subcutaneous induration AE was assigned to all 3 body areas that were treated. The same AE categorical combinations are applied by body areas as discussed above to allow for direct comparison to the categories used in the Halk meta-analysis.

**Table 3.** Adverse Event Rates per Treated Body Part of Liposuction Compared to Liposuction Followed by Helium Plasma RF

Adverse event category	Published rates for liposuction alone (*n* = 537-496,245)	Rates in abdomen/pubis (*n* = 258)	Rates in arms (*n* = 94)	Rates in back (*n* = 120)	Rates in buttocks (*n* = 27)	Rates in chest/Ax (*n* = 154)	Rates in face (*n* = 53)	Rates in hips/flanks (*n* = 231)	Rates in leg(*n* = 106	Rates in neck(*n* = 95)
Death	0%-0.06%	0%	0%	0%	0%	0%	0%	0%	0%	0%
Serious adverse events	0%-2.19%	0%	0%	0%	0%	0%	0%	0%	0%	0%
Embolism	0%-0.05%	0%	0%	0%	0%	0%	0%	0%	0%	0%
Significant bleeding	0.01%-0.23%	0%	0%	0%	0%	0%	0%	0%	0%	0%
Burn/skin Necrosis/blister	0%-2.38%	0%	0%	0%	0%	0%	0%	0%	0%	1.1% (1)
Hematoma/seroma	0.03%-35.02%	3.9% (10)	1.1% (1)	2.5% (3)	0%	3.2% (5)	0%	1.3% (3)	1.9% (2)	1.1% (1)
Infection	0.01%-0.34%	0%	0%	0%	0%	0%	0%	0%	0%	0%
Temporary nerve changes	Not reported	0%	0%	0%	0%	0%	1.9% (1)	0%	0%	1.1% (1)^a^
Wound-related problem—(scarring, inflammation, fibrosis, induration, nodule, open wound)	0.02%-1.99%	0.8% (2)	1.1% (1)	0.8% (1)	0%	0%	0%	0%	0%	3.2% (3)^a^

Note that patients may have had more than 1 area treated. ^a^These events do not represent new or elevated risks associated with the use of helium plasma RF following liposuction in the neck.

### Do the Device Settings Need to be Different for Different Body Areas?

Regarding treatment settings, the RWD in this analysis indicate that there is consistency in treatment settings used throughout the body, and that the mean values for each body area are within the treatment guidelines specified in the product IFU: 60% to 80% power and 1.5 to 3.0 lpm of helium flow (Table 4). Helium plasma RF power and helium flow data from the 483 patients included in this RWD analysis with 1184 body areas treated with liposuction followed by helium plasma RF falls within the range of IFU guidelines for the device. This demonstrates consistency in settings across all body areas.

**Table 4.** Helium Plasma RF Treatment Settings by Body Area Compared to IFU Guidelines

Body area (*n* = total number of treatments per area)	IFU guidelines power (%)	Power (%) mean ± SD	IFU guidelines helium (lpm)	Helium (lpm) mean ± SD
Abdomen and pubis (*n* = 262)	60%-80%	76.5 ± 8.9	1.5-3.0	2.6 ± 0.8
Arms (*n* = 94)	60%-80%	74.6 ± 12.4	1.5-3.0	2.8 ± 1.0
Back (*n* = 125)	60%-80%	76.6 ± 9.0	1.5-3.0	2.4 ± 1.0
Buttocks (*n* = 27)	60%-80%	75.4 ± 11.8	1.5-3.0	2.9 ± 0.9
Breast and axilla (*n* = 183)	60%-80%	74.9 ± 10.6	1.5-3.0	2.8 ± 0.7
Face (*n* = 53)	60%-80%	72.6 ± 9.7	1.5-3.0	2.6 ± 0.9
Hips/flanks (*n* = 231)	60%-80%	77.0 ± 8.8	1.5-3.0	2.5 ± 0.9
Leg (thigh, knee, calf, ankle) (*n* = 114)	60%-80%	76.4 ± 10.6	1.5-3.0	2.9 ± 0.9
Neck (*n* = 95)	60%-80%	69.9 ± 12.8	1.5-3.0	2.5 ± 0.9

IFU, instructions for use.

## DISCUSSION

**Figure 3.**
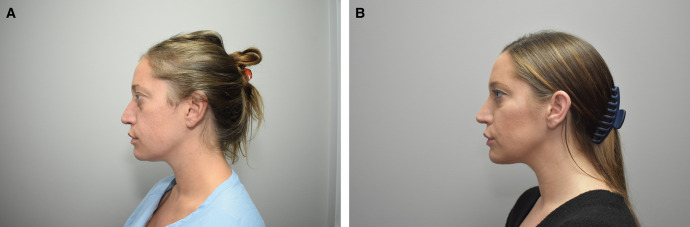
Before (A) and 9 months after (B) photographs. Thirty-one-year-old female. Helium plasma RF/liposuction to submental/neck. Helium plasma RF settings: 6J, 65%, 1.5 L, 3 passes over 13 min.

Outside of the helium plasma RF device described in this paper, there are currently no other alternative therapies—noninvasive, minimally invasive, or invasive, cleared by the FDA for use following liposuction to address excess soft-tissue laxity following a liposuction procedure. Patients who seek body contouring procedures such as liposuction desire smooth, tight skin post-recovery; however, not all patients have the skin elasticity to achieve these results and often require an additional excisional procedure to remove lax skin or are left unsatisfied due to excess skin laxity. Utilization of a device that provides energy directly to the subcutaneous connective tissue following liposuction allows the connective tissue to contract, thereby avoiding the need to perform an excisional procedure to achieve desired results (Figure 3).

No increased risks to the patients were observed with the use of helium plasma RF post-liposuction. It is interesting that temporary nerve changes were not reported by Halk et al in the published rates for liposuction alone. It is possible that this category was excluded because the focus of the publication was on more severe AEs. Temporary nerve changes are considered minor events that fully resolve over time without the need for intervention and, as such, are not typically reported as AEs by most physicians with extensive liposuction experience. Temporary impact on the MMN is a known potential risk of subcutaneous procedures in the neck because the mechanical trauma and resulting edema associated with undermining of the tissue can cause temporary impact on the MMN. Furthermore, there is known anatomical variability in the location of the MMN above and below the jawline. Temporary impact on the MMN occurred at a rate of 1.1% (1 occurrence in 95 neck treatments) in this data review. This rate was less than 6.2% (4/65) experienced in the helium plasma RF study focused on improving the appearance of lax skin in the neck and submental region.^24^ Therefore, the 1 occurrence of a temporary impact on the MMN in this data review does not represent a new or elevated risk associated with the use of helium plasma RF following liposuction when compared to liposuction alone. Additionally, aesthetic procedures, such as liposuction, involving the undermining of the skin by the placement of an instrument in the subcutaneous tissue plane, temporarily disrupt some of the sensory nerves, resulting in temporary numbness or tingling for an average of 6 to 10 weeks following the procedure with some reports of normal sensation taking 6 to 8 months to return.^25^ Sensory nerve changes occur in all areas in which the skin is undermined to perform the procedure. As part of standard clinical practice, plastic surgeons communicate this expected treatment effect to all patients undergoing liposuction or facelift procedures. Therefore, the 1 occurrence of temporary hypoesthesia/numbness in this data review does not represent a new or elevated risk associated with the use of helium plasma RF following liposuction when compared to liposuction alone.

Inclusion of subcutaneous induration and nodules in the “wound-related problem” category for the helium plasma RF data artificially inflates the rate when compared with the data from Halk et al that did not include these events in the category. Although the overall wound-related problem rate of 1.4% for liposuction followed by helium plasma RF still falls within the published range for liposuction alone, this artificial inflation of the rate does have an impact when reviewing the AE rates per body area (Table 3). The RWD for wound-related problems in the neck area in this analysis have a rate of 3.2% which is higher than the published range of 0.02% to 1.99%. However, removal of the subcutaneous induration and nodule events for the neck to allow a more direct comparison to the Halk data results in a rate of 0%.

An additional analysis was completed to compare the AE rates by body area for the procedures involving liposuction followed by helium plasma RF. These data demonstrate that there are no significant differences between the AE rates experienced in any given body area. The risks associated with the use of helium plasma RF for subcutaneous soft-tissue contraction following liposuction do not differ by body area.

As described above, the energy from the helium plasma RF device is being delivered to the connective tissue that attaches the skin to the muscle. The treatment parameters were selected to optimize the contraction of this subcutaneous soft tissue. As the connective tissue is continuous and consists primarily of collagen throughout the body,^26^ it is not necessary to change the treatment parameters based on the body area being treated. Additionally, the settings used in these areas are consistent because they are included as recommended treatment settings in the IFU and product training for the patient device.

Minimally invasive devices on the market, including laser, RF, and plasma-based devices, have the same general mechanisms of action—soft-tissue contraction through the delivery of heat to the subcutaneous tissues. The Smartlipo LAL device (Cynosure, Westford, MA) received clearance for the surgical incision, excision, vaporization, ablation, and coagulation of soft tissue.^27^ The BodyTite and FaceTite bipolar RF devices (InMode, Yokneam, Israel) received clearance for electrocoagulation and hemostasis.^28^ The ThermiX RF System (ThermiGen, Irving, TX) is indicated for use in dermatological and general surgical procedures for electrocoagulation and hemostasis.^29^ Initially, the helium plasma RF device received clearance for cutting, coagulation, and ablation of soft tissue. However, upon the submission of clinical evidence from these real-world study patients, the helium plasma RF device was able to achieve an expanded indication for contraction of subcutaneous soft tissues specifically following liposuction for aesthetic body contouring.^30^ This is the first such clearance for a minimally invasive subdermal tissue heating device. Limitations of this analysis include lack of randomization and data availability due to the nature of the retrospective design, lack of demographics in the Halk systematic review paper for comparisons of cohorts, variation in cohort sample size, lack of statistical analyses between cohorts, limited before/after photos and follow-up image time points as this was an analysis of retrospective study data where often images were not regularly collected as part of the initial studies and are not part of the private practice medical record, and lack of efficacy analyses completed as part of this analysis. Despite these limitations, this paper provides valuable safety data for the use of helium plasma RF following liposuction.

## CONCLUSION

The totality of the RWD for 483 patients and 1184 body areas is supportive of the safety of the helium plasma RF device for contracting soft tissue following liposuction in all body areas. There appears to be no new or elevated risks associated with the use of helium plasma RF following liposuction compared to liposuction alone. Additionally, these data demonstrate that there are no significant differences between the AE rates experienced in any given body area. The data further demonstrate in the real-world environment that the treatment setting guidelines outlined in the product labeling and training are followed and that treatment settings are consistent all over the body.

## Supplementary Material

ojad112_Supplementary_Data
